# Supplementary material for: Mutations in PCYT2 disrupt etherlipid biosynthesis and cause a complex hereditary spastic paraplegia
Source: Brain. 2019 Oct 22;142(11):3382–97. doi: 10.1093/brain/awz291 (PMC6821184; doi:10.1093/brain/awz291)
Supplement: awz291_Supplementary_Data [file awz291_supplementary_data.zip › awz291-Suppl_data/Supplementary_Data4.zip!/Supplementary information 4 - plasma lipidomics.zip!/Plasma lipidomics/Control vs Patient3b/Statistical plots and data/Heatmap sorted on FDR.pdf]

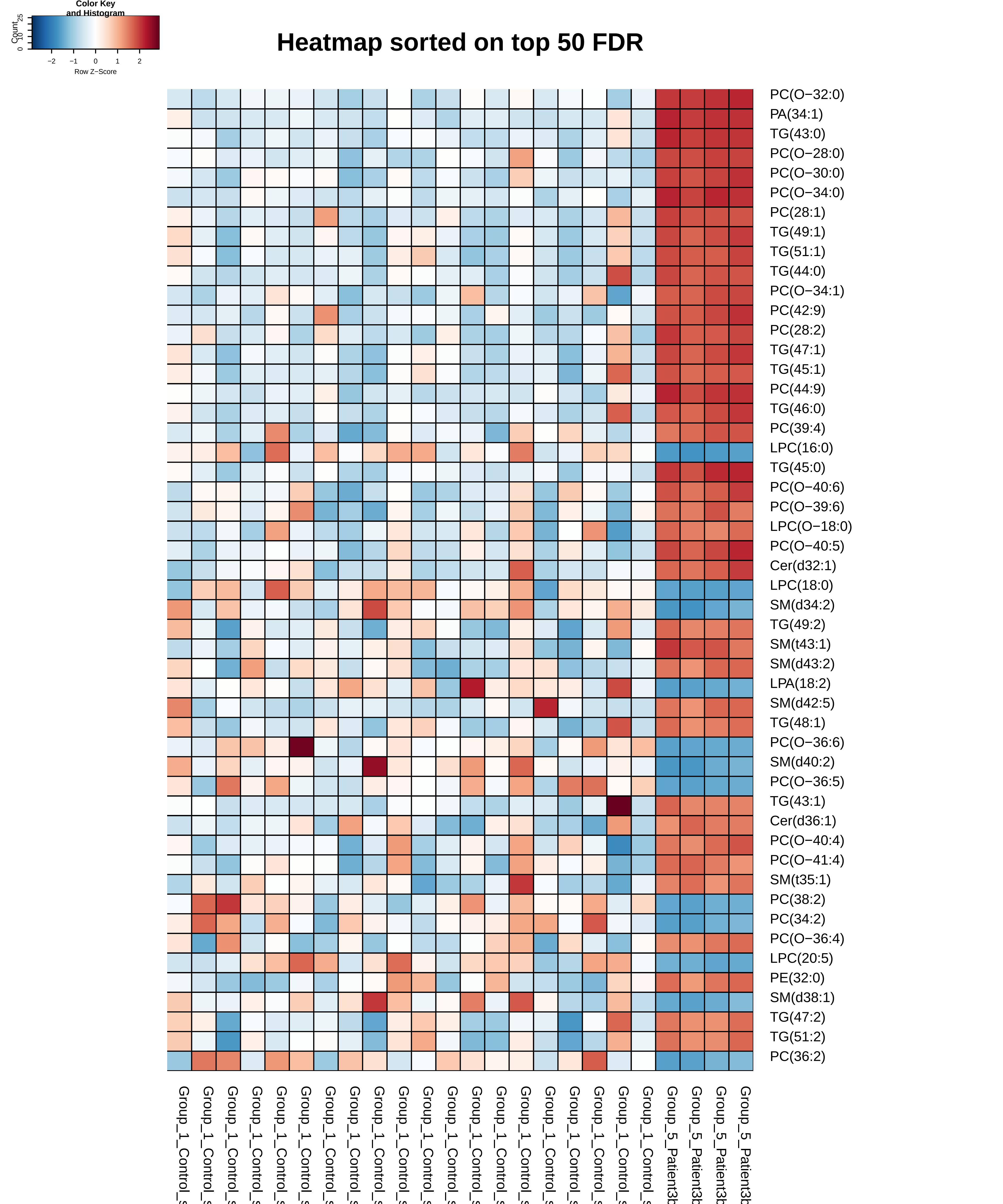

Color Key and Histogram
Count
0
10
25
−2
−1
0
1
2
Row Z−Score
Heatmap sorted on top 50 FDR
PC(O−32:0)
PA(34:1)
TG(43:0)
PC(O−28:0)
PC(O−30:0)
PC(O−34:0)
PC(28:1)
TG(49:1)
TG(51:1)
TG(44:0)
PC(O−34:1)
PC(42:9)
PC(28:2)
TG(47:1)
TG(45:1)
PC(44:9)
TG(46:0)
PC(39:4)
LPC(16:0)
TG(45:0)
PC(O−40:6)
PC(O−39:6)
LPC(O−18:0)
PC(O−40:5)
Cer(d32:1)
LPC(18:0)
SM(d34:2)
TG(49:2)
SM(t43:1)
SM(d43:2)
LPA(18:2)
SM(d42:5)
TG(48:1)
PC(O−36:6)
SM(d40:2)
PC(O−36:5)
TG(43:1)
Cer(d36:1)
PC(O−40:4)
PC(O−41:4)
SM(t35:1)
PC(38:2)
PC(34:2)
PC(O−36:4)
LPC(20:5)
PE(32:0)
SM(d38:1)
TG(47:2)
TG(51:2)
PC(36:2)
Group_1_Control_s
Group_1_Control_s
Group_1_Control_s
Group_1_Control_s
Group_1_Control_s
Group_1_Control_s
Group_1_Control_s
Group_1_Control_s
Group_1_Control_s
Group_1_Control_s
Group_1_Control_s
Group_1_Control_s
Group_1_Control_s
Group_1_Control_s
Group_1_Control_s
Group_1_Control_s
Group_1_Control_s
Group_1_Control_s
Group_1_Control_s
Group_1_Control_s
Group_5_Patient3b
Group_5_Patient3b
Group_5_Patient3b
Group_5_Patient3b
